# Association of Healthcare and Aesthetic Procedures with Infections Caused by Nontuberculous Mycobacteria, France, 2012–2020

**DOI:** 10.3201/eid2806.220520

**Published:** 2022-06

**Authors:** Kiara X. McNamara, Joseph F. Perz, Kiran M. Perkins

**Affiliations:** Centers for Disease Control and Prevention, Atlanta, Georgia, USA

**Keywords:** nontuberculous mycobacteria, bacteria, nontuberculous mycobacteria infections, tuberculosis and other mycobacteria, respiratory infections, healthcare-associated infections, aesthetic procedure‒associated infections, comparative genomics, epidemiology, case reports, retrospective study, France

**To the Editor:** Daniau et al. (*1*) described extrapulmonary nontuberculous mycobacteria (NTM) infections associated with medical procedures in France, highlighting the need for timely case reporting and genomic analysis to identify outbreak causes and prevent infections. On the basis of our experience investigating NTM healthcare-associated infections (HAIs) and outbreaks, we believe that an enhanced approach toward NTM that recognizes early signals of potential outbreaks and promptly uses the skills and investigative expertise of public health professionals is integral to mitigating disease spread. NTM pose substantial costs and burdens for patients, contributing to more hospitalizations and deaths than other waterborne pathogens (*2*)*.* Among Centers for Disease Control and Prevention consultations for waterborne HAI outbreaks, 30% were caused by NTM, accounting for 40% of cases and further substantiating need to prevent transmission in healthcare facilities (*3*). Extrapulmonary NTM infections can be challenging to detect because of their long incubation period and nonspecific signs and symptoms, which raises concern that many healthcare-associated cases are unidentified (*4*)*.*

Clinical vigilance and systematic surveillance for extrapulmonary NTM HAIs are urgently needed to detect cases, assist public health investigations, and reduce patient illness and death (*4*). Surveillance signals should trigger robust investigations, inclusive of active case-finding efforts, such as notification of potentially exposed patients, which has previously led to discovery of multiple additional cases (*3*). Investigating NTM HAIs may point to upstream causes of infection in the healthcare delivery process, such as contaminated medical products or poor infection control practices, requiring elimination of sources and appropriate interventions (*4*). Recommendations from experts and scientific evidence suggest that even a single extrapulmonary NTM HAI should prompt additional investigation *(**5*). NTM HAIs are an emerging threat to patients and carry serious consequences for patient safety. Comprehensive NTM case investigations with public health engagement are needed to inform best practices and minimize infection burdens for patients and healthcare facilities. 
